# THE LIFE STAGE AT MIGRATION MATTERS: A STUDY OF OLDER IMMIGRANTS’ SOCIAL WELLBEING BASED ON THE CLSA

**DOI:** 10.1093/geroni/igae098.3902

**Published:** 2024-12-31

**Authors:** Yeonjung Lee, Lun Li, Boah Kim

**Affiliations:** Chung-Ang University; MacEwan University, Edmonton, Alberta, Canada; Simon Fraser, Vancouver, British Columbia, Canada

## Abstract

Based on life course theory, this study examines the different social wellbeing trajectories among older immigrants in Canada based on their life stage at migration. The study applies Linear Mixed Models (LMM) to three waves of data from the Canadian Longitudinal Study on Aging (2011 to 2021). Among the sample of 4,248 older immigrants, most of them (two-thirds) migrated before the age of 30 (young adults), one-fifth migrated between 30 and 45 years old (adulthood), and about five percent of them migrated after 45 years and older (middle and older age). Results from LMM reveal that older immigrants who migrated during middle and older age reported the lowest level of social wellbeing, measured by social participation, social support, and social network size. This group of older immigrants also experienced the greatest decline in social participation, social support, and network size over ten years compared to those who migrated before 45 years old. Older adults who migrated to Canada before the age of 30 reported the best situation with higher levels of social wellbeing, and well-maintained social connection over time. In addition, employment, income level, education, and family responsibilities are significant factors related to the changes in social wellbeing over time among older immigrants. The findings from this study highlight the need to support older immigrants who come to Canada in a later life stage (i.e., after a middle-aged period), and who lack the social resources and capabilities to accumulate social capital and enhance social wellbeing.

